# Ivacaftor in a young boy with the rare gating mutation S549R - use of lung clearance index to track progress: a case report

**DOI:** 10.1186/s12890-015-0120-1

**Published:** 2015-10-16

**Authors:** Nina Lenherr, Marco Lurà, Daniel Trachsel, Philipp Latzin, Juerg Hammer

**Affiliations:** Division of Intensive Care and Pulmonology, University Children’s Hospital Basel (UKBB), University of Basel, Spitalstrasse 33, CH-4056 Basel, Switzerland

**Keywords:** Cystic fibrosis, Ivacaftor, S549R, Gating mutation, N_2_MBW, LCI

## Abstract

**Background:**

Ivacaftor acts as a potentiator of the cystic fibrosis transmembrane conductance regulator (CFTR) and increases the transepithelial chloride transport of CFTR in 9 of 10 known gating mutations causing cystic fibrosis. S549R is a rare gating mutation considered to be less sensitive to potentiators than all other gating mutations.

**Case presentation:**

We report our first experience with ivacaftor in an 8-year-old boy with the rare S549R gating mutation. Besides subjective clinical improvements, the sweat chloride level and the lung clearance index decreased impressively within a few weeks of treatment while forced expiratory volume in the first second values remained in normal range.

**Conclusion:**

We emphasize the value of measuring small airway function by lung clearance index as an outcome measure for new interventions targeting the correction of the CFTR defect at an age before traditional lung function parameters start to deteriorate.

## Background

Ivacaftor acts as a potentiator of the cystic fibrosis transmembrane conductance regulator (CFTR) and increases the transepithelial chloride (Cl^−^) transport of CFTR in 9 of 10 known gating mutations and in R117H mutation (class IV) causing cystic fibrosis. Ivacaftor has been approved in 2012 by the US Food and Drug Administration (FDA) for G551D, the most common gating mutation, after improvements in lung function and lowering of sweat chloride levels had been demonstrated in clinical trials [1]. In 2014 the approval was extended for 8 additional gating mutations, including S549R. S549R is a rare gating mutation (41 described patients in CFTR2 [2]) primarily described in the Bedouin population of the United Arab Emirates, in Saudi Arabia and in North Africa. The clinical phenotype seen with the homozygous S549R mutation is generally severe and similar to homozygous deltaF508 mutation [3, 4]. This mutation causes an additional mild processing defect besides the defective CFTR channel gating resulting in a lower in vitro response to ivacaftor than all the other gating mutations [5]. Information on clinical benefits of ivacaftor in this particularly rare mutation is scarce at present [6].

The measurement of ventilation inhomogeneity, such as the lung clearance index (LCI), is more sensitive than forced expiratory volume in the first second (FEV1) to detect early lung function abnormalities [7–10]. Further, normal tidal breathing is often easier to perform for younger children than forced expiratory maneuvers. LCI, measured by N_2_ multiple-breath washout (N_2_MBW), represents the number of lung volume turnovers required to clear the lung of N_2_ to 1/40^th^ of the starting concentration [10]. The duration of the test depends on the degree of ventilation inhomogeneity and can therefore be time-consuming in very severe lung disease. We report our first experience with ivacaftor and the usefulness of LCI in a young Swiss patient with the S549R mutation to demonstrate improvements in lung function in response to this therapy when FEV1 is within normal limits.

## Case presentation

### Case report

**Fig. 1:**
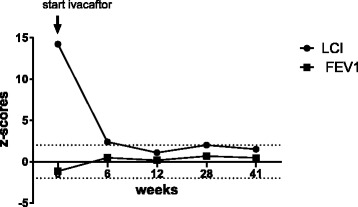
Changes in z-score of LCI and FEV1 during ivacaftor treatment

An 8-year-old boy with S549R/1717-1G > A genotype was started on ivacaftor (150 mg b.i.d.) on compassionate use. At the age of 9 months he was diagnosed with CF due to failure to thrive. His previous history was remarkable for recurring nasal polyposis requiring endoscopic surgery and exocrine pancreatic insufficiency necessitating enzyme replacement therapy. He grew with body weight and height along the 10^th^ percentile. He suffered from rather mild respiratory symptoms, primarily intermittent productive cough, and had close to normal lung function parameters in previous years as measured by body plethysmography and spirometry (minimal z-score of FEV1: −1.2). Sputum cultures grew *Haemophilus influenzae* and *Staphylococcus aureus* on several occasions. After 6 weeks of ivacaftor treatment, the patient reported clinical improvements in cough frequency, sputum production, physical performance, and less salt cravings. He gained 1.4 kg in body weight without changing the dose of his pancreatic enzyme replacement therapy. His sweat chloride level (Macroduct®) decreased from 115 mmol/l before ivacaftor to 40 mmol/l after 6 weeks and 52 mmol/l after 41 weeks (normal < 30 mmol/l [11]) of treatment. His FEV1 increased from 1.25 L (−1.2 z-score) to 1.65 L (+0.5 z-score) after 41 weeks of ivacaftor therapy. The LCI (normal < 8) measured by N_2_-MBW decreased from 14.5 to 8.3 after 6 weeks and 7.8 after 41 weeks of ivacaftor treatment (Table 1 and Fig. 1).

**Table 1:** Improvement of functional parameters during ivacaftor therapy

	Start	6 weeks	12 weeks	28 weeks	41 weeks
Sweat chloride level [mmol/l]	115	40	30	40	52
FEV1 [l ] (z-score) ^a^	1.25 (−1.2)	1.53 (0.5)	1.48 (0.2)	1.63 (0.7)	1.65 (0.5)
LCI (z-score) ^b^	14.5 (14.2)	8.3 (2.4)	7.6 (1.1)	8.1 (2.0)	7.8 (1.5)
Weight [kg] (z-score)	21.8 (−1.5)	23.2 (−1.1)	23.7 (−0.9)	23.9 (−0.9)	26 (−0.5)
BMI [kg/m^2^] (z-score)	14.6 (−0.8)	15.6 (−0.2)	15.7 (−0.1)	15.5 (−0.2)	16.4 (0.2)

Improvement of different functional parameters during 41 weeks of ivacaftor therapy: *FEV1* Forced expiratory volume in the first second, *LCI* lung clearance index. ^a^Reference population for z-score values of FEV1 are the global multi-ethnic reference equations of the global lung function initiative [16]. ^b^Reference population for z-score values of the LCI is our intracentric age-matched healthy control population

### Discussion

Ivacaftor is the first authorized drug that improves defective CFTR function in rare mutations by potentiating the CFTR channel gating function. In vitro data suggests that ivacaftor has a similar effect on 9 of 10 CFTR gating mutations. However, the weakest drug effect was described for the S549R mutation [5]. S549R was initially considered a class II mutation leading to defective CFTR protein processing [12]. Recent electrophysiological studies using Fischer rat thyroid cells have suggested the presence of a predominant gating defect besides a mild processing defect of CFTR in S549R [5]. The processing defect may account for the lower ivacaftor response observed in vitro. Against this background, the impressive clinical and functional improvement in our patient with the S549R mutation was better than expected. These results are in line with a recently published clinical trial study including four patients with S549R mutation [6].

One limitation of this report is that no nasal potential differences or intestinal current measurement have been performed as further functional parameters. The benefit of using LCI as an outcome measure should not be over generalised from this case. However this report highlights the additional value of LCI as a sensitive parameter in relation to the traditional lung function parameter FEV1. Other studies have shown that the LCI of N_2_MBW is particularly useful in monitoring the early course of lung disease in young children with CF, particularly in those with normal spirometry [13, 14]. The LCI is already elevated in presymptomatic or minimally symptomatic infants and young children with CF [9]. In our patient, the LCI decreased after 41 weeks of ivacaftor treatment from a z-score of 14.2 to 1.5, while the z-score for FEV1 remained within the normal range (z-score −1.2 to 0.5). This is in agreement with previous work indicating that LCI is a more sensitive parameter to detect treatment success in young CF patients compared to spirometry, especially in children with little respiratory symptoms and near-normal spirometric lung volumes such as FEV1 [15].

## Conclusion

This report provides anecdotal evidence of benefit of ivacafor in S549R mutation.

Further it illustrates the potential value of lung clearance index to serve as an outcome measure for new interventions targeting the correction of the CFTR defect at an early stage of the disease. This is relevant since ivacaftor approval has recently been extended to preschool children where performance and interpretation of spirometry is even more challenging. Such measurements may help to convince medical healthcare payers to cover the cost of the drug in our young CF population.

## Consent

Written informed consent was obtained from the parents of the patient for publication of this case report and any accompanying images. A copy of the written consent is available for review by the Editor of this journal.

## Abbreviations

CFTR: Cystic fibrosis transmembrane conductance regulator
Cl^−^: Chloride
FDA: Food and drug administration
FEV1: Forced expiratory volume in the first second
LCI: Lung clearance index
N_2_MBW: Nitrogen multiple-breath washout
